# Supplementary material for: Multistep antimicrobial stewardship intervention on antibiotic prescriptions and treatment duration in children with pneumonia
Source: PLoS One. 2021 Oct 27;16(10):e0257993. doi: 10.1371/journal.pone.0257993 (PMC8550372; doi:10.1371/journal.pone.0257993)
Supplement: S1 Table — (DOCX) [file pone.0257993.s001.docx]

**Supporting Information**

**S1 Table.** Interrupted time series model parameters for outpatients and inpatients.

|  | Estimate | Standard  Error | z | P-value | Relative Risk | Lower limit (2.5%) | Upper limit (97.5%) |
| --- | --- | --- | --- | --- | --- | --- | --- |
| Outpatients |  |  |  |  |  |  |  |
| (Intercept) | -0.546 | 0.153 | -3.566 | 0.000 | 0.579 | 0.429 | 0.782 |
| 2015-CP | -0.650 | 0.301 | -2.156 | **0.031** | **0.522** | **0.289** | **0.943** |
| 2019-CP | -0.708 | 0.541 | -1.310 | 0.190 | 0.492 | 0.171 | 1.422 |
| Time in bimesters | -0.029 | 0.030 | -0.955 | 0.340 | 0.972 | 0.916 | 1.031 |
