# Supplementary material for: Multistep antimicrobial stewardship intervention on antibiotic prescriptions and treatment duration in children with pneumonia
Source: PLoS One. 2021 Oct 27;16(10):e0257993. doi: 10.1371/journal.pone.0257993 (PMC8550372; doi:10.1371/journal.pone.0257993)
Supplement: S2 Table — The reference for all the models is the Pre period. Significant P values are in bold. Quasi significant p values are in italic. (DOCX) [file pone.0257993.s002.docx]

**Supporting Information**

**S2 Table** Relative risk with 95% confidence intervals and p-value of univariate regression models for DOT, LOT, DOT/LOT ratios and LOS. The reference for all the models is the Pre period. Significant P values are in bold. Quasi significant p values are in italic.

|  | Inpatient | | Outpatients | |
| --- | --- | --- | --- | --- |
|  | RR (95% CI) | P | RR (95% CI) | P |
| DOT |  |  |  |  |
| Post1 | 0.564 (0.355-0.897) | **0.015** | 0.770 (0.716-0.829) | **<0.001** |
| Post2 | 0.782 (0.512-1.193) | 0.254 | 0.713 (0.669-0.761) | **<0.001** |
| Post3 | 0.600 (0.394-0.914) | **0.017** | 0.749 (0.703-0.799) | **<0.001** |
| Post4 | 0.716 (0.464-1.104) | 0.130 | 0.747 (0.701-0.795) | **<0.001** |
| Post5 | 0.569 (0.322-1.005) | *0.052* | 0.793 (0.720-0.872) | **<0.001** |
| Post6 | 0.537 (0.317-0.908) | **0.020** | 0.725 (0.679-0.775) | **<0.001** |
| LOT |  |  |  |  |
| Post1 | 0.760 (0.563-1.026) | *0.073* | 0.845 (0.798-0.895) | **<0.001** |
| Post2 | 0.950 (0.722-1.250) | 0.716 | 0.801 (0.762-0.842) | **<0.001** |
| Post3 | 0.813 (0.619-1.068) | 0.137 | 0.836 (0.796-0.879) | **<0.001** |
| Post4 | 0.880 (0.664-1.165) | 0.372 | 0.824 (0.784-0.865) | **<0.001** |
| Post5 | 0.798 (0.552-1.154) | 0.231 | 0.855 (0.794-0.922) | **<0.001** |
| Post6 | 0.721 (0.513-1.013) | *0.060* | 0.816 (0.776-0.859) | **<0.001** |
| DOT/LOT |  |  |  |  |
| Post1 | 0.742 (0.621-0.886) | **0.001** | 0.911 (0.829-1002) | *0.056* |
| Post2 | 0.823 (0.711-0.952) | **0.009** | 0.890 (0.819-0.968) | **0.007** |
| Post3 | 0.738 (0.633-0.860) | **<0.001** | 0.896 (0.825-0.972) | **0.009** |
| Post4 | 0.813 (0.698-0.948) | **0.008** | 0.906 (0.836-0.983) | **0.017** |
| Post5 | 0.713 (0.567-0.896) | **0.004** | 0.927 (0.818-1.050) | 0.234 |
| Post6 | 0.745 (0.603-0.920) | **0.006** | 0.889 (0.816-0.968) | **0.007** |
| LOS |  | |  |  |
| Post1 | 0.585 (0.357-0.959) | **0.033** |  |  |
| Post2 | 0.786 (0.501-1.233) | 0.295 |  |  |
| Post3 | 0.595 (0.380-0.931) | **0.023** |  |  |
| Post4 | 0.676 (0.427-1.072) | 0.096 |  |  |
| Post5 | 0.621 (0.339-1.138) | 0.123 |  |  |
| Post6 | 0.611 (0.349-1.069) | *0.084* |  |  |
