# Supplementary material for: Multistep antimicrobial stewardship intervention on antibiotic prescriptions and treatment duration in children with pneumonia
Source: PLoS One. 2021 Oct 27;16(10):e0257993. doi: 10.1371/journal.pone.0257993 (PMC8550372; doi:10.1371/journal.pone.0257993)
Supplement: S1 File — (DOCX) [file pone.0257993.s003.docx]

**Supporting Information**

**S1 File. CAP-ICD9 analysis**

Inpatients

In total 157 out of 728 CAP-ICD9 episodes were included in the inpatients group; the demographic characteristics of children included were similar with respect to sex and age (**Table 1a**). No difference in positivity for Mycoplasma pneumoniae by serology or blood test was noted.

All CAP-ICD9 episodes except one received antibiotic treatment. There was a significant variation in the BS-AT rate, from 100% in the Pre period to around 60% in the Post1-, Post2-, Post3-, Post4-, Post5- periods. After the introduction of 2019-CP (Post6), the BS-AT rate decreased to 38.5%.

The rate of macrolides treatments decreased significantly (p value <0.001) from 66.7% before 2015-CP implementation to 0% after 2019-CP implementation. Cephalosporins use decreased as well (from 77.8% Pre to 47.6% Post1, 57.6% Post2, 44.1% Post3, 31% Post4, 50% Post5, 38.5% Post6), but the difference was not statistically significant (**Table 2a**).

With a variation from 13.5 to 9.0 to 7.0 days, DOT decreased significantly in the periods after 2015-CP implementation, with the Post6-period having the lowest difference from the Pre-period (-49.4%). (**Table 2a**, **Table 5a**)

LOT was 27.3% lower in the Post1 period and 29.5% and 31.4% lower in the Post5- and Post6-periods with respect to Pre-period. DOT/LOT ratio was around 30% lower in the Post-periods compared to the Pre. LOS was 34.4%, 32.1% lower in the Post-1, Post3-period compared to the Pre-period; LOS in the Post6-period was quasi-significantly lower than Pre-period by 32.7%. (**Table 5a**)

Outpatients

Outpatients with CAP-ICD9 included in the study were 446; the demographic characteristics of children included were similar with respect to sex and age (**Table 3a**). Mycoplasma pneumoniae was found just in one case in the Pre-period. Chest X-ray performed rates varied from a minimum of 41.7% in the Post3 to a maximum of 95.2% in the Post5-period.

All CAP-ICD9 episodes except three received antibiotic treatment. BS-AT rate varied significantly (p<0.001), from 50% in the Pre-period to 20.3% in the Post6-period. After the introduction of 2019-CP (Post6), the BS-AT rate decreased to 20.3%. (**Table4a**)

The cephalosporins and macrolides treatments rate varied significantly from 19.6% to 5.8% and from 25.0% to 10.1% in the Pre- and Post6-period, respectively. (**Table 4a**)

DOT decreased from 9.0 days (IQR:0.5) to 7.0 days (IQR:1.0) after 2015-CP implementation and from 9.0 days (IQR:5.3) to 7.0 (IQR:5.0) to a minimum of 6.0 (IQR:1.0) in the Post2-period; DOT were about 20% lower in the different post periods compared to the Pre-period (**Table 4a** and **Table 5a**). A similar decrease was noted in LOT where median values varied from 9.0 to 7.0 after 2015-CP implementation. (**Table 4a** and **Table 5a**)

DOT/LOT ratios were significantly lower in all periods post 2015-CP implementation compared to the pre-implementation ratio except for Post1 and Post5 periods. (**Table 5a**)

**Tables**

Table 1a. Demographic characteristics of patients hospitalized for CAP-ICD9 in different periods. Only significant p-values are reported

|  | **Pre** | **Post1** | **Post2** | **Post3** | **Post4** | **Post5** | **Post6** | **p-value** |
| --- | --- | --- | --- | --- | --- | --- | --- | --- |
| **N of episodes** | 18 | 21 | 33 | 33 | 29 | 10 | 13 |  |
| **Age, months, Median (IQR)** | 34.0 (30.3) | 39 (37.0) | 40 (69) | 29 (53) | 30 (40) | 20.5 (14.5) | 25 (16) |  |
| **Age class, 3-35 months, N, (%)** | 10 (55.6) | 8 (38.1) | 16 (48.5) | 18 (54.5) | 16 (55.2) | 8 (80.0) | 10 (76.9) |  |
| **Sex, (%), male** | 13 (72.2) | 9 (42.9) | 21 (63.6) | 17 (51.5) | 12 (41.4) | 8 (80.0) | 6 (46.2) |  |
| **Body weight, kg, Median (IQR)** | 13.5 (5.0) | 13.0 (7.5) | 14.0 (11.8) | 13.0 (8.9) | 13.0 (6.0) | 12.0 (1.8) | 11.0 (4.5) |  |
| **Vaccination, Yes, (%)** | 17 (94.4) | 20 (95.2) | 32 (97.0) | 33 (100.0) | 29 (100.0) | 10 (100.0) | 12 (92.3) |  |
| Not completed the immunization plan | 0 (0.0) | 0 (0.0) | 6 (18.8) | 3 (9.1) | 1 (3.5) | 1 (10.0) | 1 (5.0) | 0.014 |
| **C reactive protein exam, Yes, (%)** | / | / | / | / | 29 (100.0) | 10 (100.0) | 13 (100.0) |  |
| mg/L, Median (IQR) | / | / | / | / | 47.3 (181.3) | 189.5 (168.3) | 30.0 (20.3) |  |
| **Procalcitonin, Yes, (%)** | / | / | / | / | 9 (31.0) | 5 (50.0) | 13 (100.0) |  |
| ug/L, Median [Min, Max] | / | / | / | 0.33 (132.5) | 2.43 (3.63) | 3.75 (4.04) | 1.40 (2.68) |  |
| **M.Pneumoniae positivity** | 3 (16.7) | 2 (9.5) | 1 (3.0) | 2 (6.1) | 4 (13.8) | 0 (0.0) | 0 (0.0) |  |
| **Chest X-ray exams, Yes, (%)** | 17 (94.4) | 21 (100.0) | 31 (93.9) | 31 (93.9) | 28 (96.6) | 10 (100.0) | 13 (100.0) |  |
| **Antibiotic administered** | 18 (100.0) | 21 (100.0) | 33 (100.0) | 33 (100.0) | 28 (96.6) | 10 (100.0) | 13 (100.0) |  |

Table 2a Antibiotic treatments for patients hospitalized with CAP-ICD9. Only significant p-values are reported

|  | | **Pre** | | **Post1** | | **Post2** | | **Post3** | | **Post4** | | **Post5** | **Post6** | | **p-value** | |
| --- | --- | --- | --- | --- | --- | --- | --- | --- | --- | --- | --- | --- | --- | --- | --- | --- |
| **N of episodes with a prescription** | | 18 | | 21 | | 33 | | 33 | | 28 | | 10 | 13 | |  | |
| Amoxicillin, N, (%) | | 6 (33.3) | | 16 (76.2) | | 14 (42.4) | | 17 (51.5) | | 17 (60.7) | | 7 (70.0) | 8 (61.5) | |  | |
| Amikacin, N, (%) | | 0 (0.0) | | 0 (0.0) | | 0 (0.0) | | 0 (0.0) | | 1 (3.6) | | 0 (0.0) | 0 (0.0) | |  | |
| Co-amoxiclav, N, (%) | | 2 (11.1) | | 1 (4.8) | | 7 (21.2) | | 8 (24.2) | | 5 (17.9) | | 0 (0.0) | 3 (23.1) | |  | |
| Beta-lactams inhibitors, N, (%) | | 4 (22.2) | | 1 (4.8) | | 7 (21.2) | | 8 (24.2) | | 5 (17.9) | | 0 (0.0) | 3 (23.1) | |  | |
| Cephalosporins, N, (%) | | 14 (77.8) | | 10 (47.6) | | 19 (57.6) | | 14 (42.4) | | 9 (32.1) | | 5 (50.0) | 5 (38.5) | |  | |
| Macrolides, N, (%) | | 12 (66.7) | | 7 (33.3) | | 5 (15.2) | | 6 (18.2) | | 9 (32.1) | | 1 (10.0) | 0 (0.0) | | < 0.001 | |
| Glycopeptides, N, (%) | | 4 (22.2) | | 0 (0.0) | | 2 (6.1) | | 1 (3.0) | | 1 (3.6) | | 0 (0.0) | 0 (0.0) | | 0.033 | |
| Clindamycin, N, (%) | | 0 (0.0) | | 1 (4.8) | | 4 (12.1) | | 5 (15.2) | | 2 (7.1) | | 2 (20.0) | 1 (7.7) | |  | |
| **Broad-spectrum treatment, N, (%)** | | 18 (100.0) | | 14 (66.7) | | 23 (69.7) | | 19 (57.6) | | 17 (60.7) | | 6 (60.0) | 5 (38.5) | | 0.006 | |
| **DOT, Mean (SD)** | | 17.1 (9.6) | | 9.6 (5.2) | | 13.3 (13.7) | | 10.2 (5.4) | | 12.2 (10.0) | | 9.7 (6.1) | 9.2 (6.2) | |  | |
| **DOT, Median (IQR)** | | 13.5 (9.3) | | 9.0 (3.0) | | 9.0 (3.0) | | 9.0 (3.0) | | 9.0 (4.8) | | 9.0 (5.3) | 7.0 (5.0) | |  | |
| **LOT, Mean (SD)** | | 10.78 (4.05) | | 8.19 (3.12) | | 10.24 (5.99) | | 8.73 (3.52) | | 9.61 (5.32) | | 8.60 (4.2) | 7.77 (3.32) | |  | |
| **LOT, Median (IQR)** | | 9.5 (1.8) | | 9.0 (3.0) | | 9.0 (3.0) | | 8.0 (3.0) | | 8.5 (3.3) | | 9.0 (3.5) | 7.0 (4.0) | |  | |
| **DOT/LOT, Mean (SD)** | | 1.53 (0.38) | | 1.14 (0.26) | | 1.17 (0.34) | | 1.17 (0.33) | | 1.22 (0.32) | | 1.09 (0.18) | 1.12 (0.28) | |  | |
| **DOT/LOT, Median (IQR)** | | 1.5 (0.6) | | 1.0 (0.3) | | 1.0 (0.0) | | 1.0 (0.2) | | 1.0 (0.5) | | 1.0 (0.0) | 1.0 (0.0) | |  | |
| **LOS, Mean (SD)** | 8.06 (7.73) | | 4.71 (2.92) | | 6.33 (6.50) | | 4.76 (3.07) | | 5.45 (4.12) | | 5.00 (2.26) | | | 4.92 (3.07) | | |
| **LOS, Median (IQR)** | 5.0 (3.8) | | 4.0 (2.0) | | 4.0 (3.0) | | 4.0 (2.0) | | 4.0 (2.3) | | 4.5 (1.8) | | | 4.0 (3.0) | |  |

Table 3a. Demographic characteristics of outpatients with CAP-ICD9 in different periods. Only significant p-values are reported

|  | **Pre** | **Post1** | **Post2** | **Post3** | **Post4** | **Post5** | **Post6** | **p-value** |
| --- | --- | --- | --- | --- | --- | --- | --- | --- |
| **N of episodes** | 56 | 41 | 102 | 72 | 85 | 21 | 69 |  |
| **Age, months, Median (IQR)** | 44.50(31.25) | 37.00(20.00) | 38.50(33.00) | 35.00(33.25) | 42.00(32.00) | 34.00(62.00) | 44.00(53.00) |  |
| **Age class, 3-35 months, (%)** | 20(35.7) | 18(43.9) | 44(43.1) | 36(50.0) | 35(41.2) | 11(52.4) | 28(40.6) |  |
| **Sex, male, (%)** | 24(42.9) | 18(43.9) | 56(54.9) | 37(51.4) | 52(61.2) | 12(57.1) | 41(59.4) |  |
| **Bodyweight, kg, Median (IQR)** | 15.00(7.38) | 14.00(4.70) | 15.00(8.50) | 14.00(6.57) | 15.00(6.00) | 15.00(11.00) | 15.00(10.00) | 0.036 |
| **Vaccination, Yes, (%)** | 54(96.4) | 39(95.1) | 95(93.1) | 69(95.8) | 83(97.6) | 20(95.2) | 67(97.1) |  |
| Not completed the immunization plan, (%) | 2(3.7) | 0(0.0) | 1(1.1) | 4(5.8) | 4(4.8) | 2(10.0) | 3(4.5) |  |
| **C reactive protein exam, Yes, (%)** | / | / | / | / | 19(22.4) | 7(33.3) | 14(20.3) |  |
| mg/L, Median (IQR) | / | / | / | / | 54.20(92.60) | 38.00(118.10) | 20.65(87.30) |  |
| **Procalcitonin, Yes, (%)** | / | / | / | / | 2(2.4) | 1(4.8) | 7(10.1) |  |
| ug/L, Median (IQR) | / | / | / |  | 9.02(5.79) | 1.09(0.00) | 0.13(0.79) |  |
| **M.Pneumoniae positivity** | 1(1.8) | 0(0.0) | 0(0.0) | 0(0.0) | 0(0.0) | 0(0.0) | 0(0.0) |  |
| **Chest X-ray exams, Yes, (%)** | 27(48.2) | 19(46.3) | 48(47.1) | 30(41.7) | 49(57.6) | 20(95.2) | 43(62.3) | <0.001 |
| **Antibiotic administered, Yes, (%)** | 56(100.0) | 41(100.0) | 102(100.0) | 71(98.6) | 84(98.8) | 20(95.2) | 69(100.0) |  |

Table 4a Antibiotic treatments for outpatients with CAP-ICD9. Only significant p-values are reported

|  | **Pre** | **Post1** | **Post2** | **Post3** | **Post4** | **Post5** | **Post6** | **p-value** |
| --- | --- | --- | --- | --- | --- | --- | --- | --- |
| **N of episodes with a prescription** | 56 | 41 | 102 | 71 | 84 | 20 | 69 |  |
| Amoxicillin, N, (%) | 37 (66.1%) | 32 (78.0%) | 86 (84.3%) | 59 (83.1%) | 65 (77.4%) | 14 (70.0%) | 55 (79.7%) |  |
| Amikacin, N, (%) | 0 (0.0%) | 0 (0.0%) | 0 (0.0%) | 0 (0.0%) | 1 (1.2%) | 0 (0.0%) | 0 (0.0%) |  |
| Co-amoxiclav, N, (%) | 9 (16.1%) | 7 (17.1%) | 10 (9.8%) | 9 (12.7%) | 8 (9.5%) | 4 (20.0%) | 4 (5.8%) |  |
| Beta-lactams inhibitors, N, (%) | 9 (16.1%) | 7 (17.1%) | 10 (9.8%) | 9 (12.7%) | 8 (9.5%) | 4 (20.0%) | 4 (5.8%) |  |
| Cephalosporins, N, (%) | 11 (19.6%) | 4 (9.8%) | 6 (5.9%) | 4 (5.6%) | 5 (6.0%) | 0 (0.0%) | 4 (5.8%) | 0.022 |
| Macrolides, N, (%) | 14 (25.0%) | 3 (7.3%) | 2 (2.0%) | 2 (2.8%) | 9 (10.7%) | 3 (15.0%) | 7 (10.1%) | < 0.001 |
| Glycopeptides, N, (%) | 0 (0.0%) | 0 (0.0%) | 0 (0.0%) | 0 (0.0%) | 0 (0.0%) | 0 (0.0%) | 0 (0.0%) | / |
| Clindamycin, N, (%) | 0 (0.0%) | 0 (0.0%) | 0 (0.0%) | 0 (0.0%) | 0 (0.0%) | 0 (0.0%) | 0 (0.0%) | / |
| **Broad-spectrum treatment, yes, (%)** | 28 (50.0%) | 11 (26.8%) | 18 (17.6%) | 15 (21.1%) | 20 (23.8%) | 7 (35.0%) | 14 (20.3%) | < 0.001 |
| **DOT, Mean (SD)** | 9.93 (4.20) | 7.29 (2.26) | 6.54 (1.13) | 6.97 (1.19) | 7.17 (1.94) | 7.95 (2.74) | 6.81 (1.67) |  |
| **DOT, Median (IQR)** | 9.00(0.50) | 7.00(1.00) | 6.00(1.00) | 7.00(1.00) | 7.00(0.00) | 7.00(2.00) | 7.00(1.00) |  |
| **LOT, Mean (SD)** | 8.59 (1.93) | 6.88 (1.17) | 6.50 (1.08) | 6.89 (0.92) | 6.96 (1.42) | 7.50 (1.43) | 6.78 (1.66) |  |
| **LOT, Median (IQR)** | 9.00(0.25) | 7.00(1.00) | 6.00(0.75) | 7.00(1.00) | 7.00(0.00) | 7.00(2.00) | 7.00(1.00) |  |
| **DOT/LOT Ratio, Mean (IQR)** | 1.15 (0.32) | 1.05 (0.18) | 1.01 (0.07) | 1.01 (0.08) | 1.03 (0.14) | 1.05 (0.22) | 1.00 (0.04) |  |
| **DOT/LOT Ratio, Median (IQR)** | 1.0 (0.0) | 1.0 (0.0) | 1.0 (0.0) | 1.0 (0.0) | 1.0 (0.0) | 1.0 (0.0) | 1.0 (0.0) |  |

Table 5a Relative risk with 95% confidence intervals and p-value of univariate regression models for DOT, LOT, DOT/LOT ratios, LOS and BS-AT. The reference for all the models is the Pre period. Significant P values are in bold. Quasi significant p-values are in italic.

|  | Inpatients | | Outpatients | |
| --- | --- | --- | --- | --- |
|  | RR (95% CI) | P | RR (95% CI) | P |
| DOT |  |  |  |  |
| Post1 | 0.564 (0.355-0.897) | **0.016** | 0.735 (0.659-0.818) | **<0.001** |
| Post2 | 0.782 (0.512-1.194) | 0.254 | 0.659 (0.604-0.719) | **<0.001** |
| Post3 | 0.599 (0.392-0.914) | **0.018** | 0.702 (0.639-0.771) | **<0.001** |
| Post4 | 0.729 (0.471-1.128) | 0.155 | 0.722 (0.659-0.790) | **<0.001** |
| Post5 | 0.569 (0.322-1006) | *0.052* | 0.801 (0.698-0.918) | **0.001** |
| Post6 | 0.537 (0.317-0.908) | **0.020** | 0.686 (0.624-0.754) | **<0.001** |
| LOT |  |  |  |  |
| Post1 | 0.760 (0.562-1.027) | *0.074* | 0.801 (0.741-0.865) | **<0.001** |
| Post2 | 0.950 (0.722-1.251) | 0.716 | 0.757 (0.711-0.806) | **<0.001** |
| Post3 | 0.810 (0.615-1.066) | 0.132 | 0.802 (0.750-0.858) | **<0.001** |
| Post4 | 0.891 (0.672-1.183) | 0.426 | 0.811 (0.760-0.865) | **<0.001** |
| Post5 | 0.798 (0.551-1.155) | 0.231 | 0.873 (0.791-0.963) | **0.007** |
| Post6 | 0.721 (0.512-1.014) | *0.060* | 0.790 (0.738-0.845) | **<0.001** |
| DOT/LOT |  |  |  |  |
| Post1 | 0.742 (0.621-0.886) | **0.001** | 0.917 (0.797-1.056) | 0.228 |
| Post2 | 0.823 (0.711-0.952) | **0.009** | 0.870 (0.778-0.974) | **0.016** |
| Post3 | 0.739 (0.633-0.863) | **<0.001** | 0.876 (0.776-0.988) | **0.032** |
| Post4 | 0.818 (0.701-0.953) | **0.010** | 0.890 (0.793-0.999) | **0.048** |
| Post5 | 0.713 (0.567-0.896) | **0.004** | 0.917 (0.769-1.094) | 0.335 |
| Post6 | 0.745 (0.603-0.920) | **0.006** | 0.869 (0.768-0.982) | **0.025** |
| LOS |  |  |  |  |
| Post1 | 0.585 (0.357-0.960) | **0.034** |  |  |
| Post2 | 0.786 (0.500-1.235) | 0.297 |  |  |
| Post3 | 0.591 (0.376-0.928) | **0.022** |  |  |
| Post4 | 0.687 (0.431-1.095) | 0.114 |  |  |
| Post5 | 0.621 (0.338-1.140) | 0.124 |  |  |
| Post6 | 0.611 (0.349-1.071) | *0.085* |  |  |
